# OCTUNE: Optimal Control Tuning Using Real-Time Data with Algorithm and Experimental Results

**DOI:** 10.3390/s22239240

**Published:** 2022-11-28

**Authors:** Mohamed Abdelkader, Mohamed Mabrok, Anis Koubaa

**Affiliations:** 1 College of Computer & Information Sciences, Robotics & Internet of Things Laboratory, Prince Sultan University, P.O. Box 66833, Riyadh 11586, Saudi Arabia; 2 Mathematics Program, Department of Mathematics, Statistics and Physics, College of Arts and Sciences, Qatar University, Doha P.O. Box 2713, Qatar

**Keywords:** robotics, unmanned aerial vehicles, control tuning, open-source

## Abstract

Autonomous robots require control tuning to optimize their performance, such as optimal trajectory tracking. Controllers, such as the Proportional–Integral–Derivative (PID) controller, which are commonly used in robots, are usually tuned by a cumbersome manual process or offline data-driven methods. Both approaches must be repeated if the system configuration changes or becomes exposed to new environmental conditions. In this work, we propose a novel algorithm that can perform online optimal control tuning (OCTUNE) of a discrete linear time-invariant (LTI) controller in a classical feedback system without the knowledge of the plant dynamics. The OCTUNE algorithm uses the backpropagation optimization technique to optimize the controller parameters. Furthermore, convergence guarantees are derived using the Lyapunov stability theory to ensure stable iterative tuning using real-time data. We validate the algorithm in realistic simulations of a quadcopter model with PID controllers using the known Gazebo simulator and a real quadcopter platform. Simulations and actual experiment results show that OCTUNE can be effectively used to automatically tune the UAV PID controllers in real-time, with guaranteed convergence. Finally, we provide an open-source implementation of the OCTUNE algorithm, which can be adapted for different applications.

## 1. Introduction

Control tuning is a fundamental concept in any control system’s design cycle; see, for instance, Refs. [1,2] and the references therein. In particular, robotic systems require control tuning to perform different levels of autonomous tasks with the desired performance. In these systems, conventional controllers, such as the Proportional–Integral–Derivative (PID) controller, are usually tuned using an iterative manual process or offline data-driven methods.

For instance, in quadrotor control, known open-source autopilots, such as PX4 [3] and Ardupilot [4], use either manual tuning or non-optimal auto-tuning methods. Furthermore, many dynamical systems exhibit complex characteristics, such as non-linearity, time-varying parameters, and time delay, which leads to different operating conditions and/or disturbances, leading to poor control.

Generally speaking, control tuning methodologies can be classified as offline methods and online methods. In the offline methods, such as linear–quadratic Gaussian control (LQG) [5,6] and H-infinity control [7], the controller requires an accurate model of the system dynamics under control. The controller is designed for the model of the system and is tuned before the implementation stage. These controllers work well with systems that have an accurate linear model. However, they give poor performance otherwise. On the other hand, in the online adaptive methods, the model is often required as well. However, the controller can adapt to the un-modeled system dynamics, which is well-known under the adaptive control theory [8,9], which is well-developed and established in linear and nonlinear control systems.

Online model-free methods also exist in several studies [10,11,12]. Another class of gradient-descent-based algorithms also exist in the literature. For instance, a control method based on an adaptive PID neural network and particle swarm optimization (PSO) algorithm was developed in [13]. In [14], the investigation of adaptive learning control for underwater vehicles (AUVs) with unknown uncertainties using gradient descent algorithm is presented, where the unknown nonlinear functions in the system are approximated by radial basis function neural networks.

In [15], another adaptive gradient descent algorithm combined with a fuzzy system was developed to improve the attitude estimation accuracy and adaptability of unmanned underwater vehicles under various ocean environments. Many attempts have been made to build auto-tuned PID controllers using different adaptive learning techniques [16]. For instance, the authors in [17,18] used genetic algorithms to tune a PID. Furthermore, the use of a neural network to tune a PID controller through extensive training was discussed in [19]. However, these techniques have several drawbacks, such as a lack of stability guarantees, slow convergence, or implementation constraints.

In this paper, we develop an online learning algorithm based on the backpropagation learning technique to learn a controller for a dynamical system without knowing the system model. Our control-learning algorithm is based on the work presented in [20,21], where the backpropagation learning technique is used in system identification for linear dynamical models. The use of backpropagation learning techniques in training systems is becoming the norm due to the extensive use of backpropagation algorithms in the modern machine-learning domain. The accessibility of the backpropagation algorithms in several software packages, such as TensorFlow [22] and PyTorch [23], has made them more attractive and easy to use.

The backpropagation learning technique was used in several attempts in PID tuning. For instance, in [24], a fuzzy PID controller, which is a combination of a fuzzy controller with a PID neural network (PIDNN), was proposed. In [25], a conventional Neuro PID controller for linear or nonlinear systems that was unaffected by the unpredictability of the system’s parameters and disturbances, such as noise, was developed. However, again, these methods require a model for the controlled system as they lack stability guarantees.

This paper proposes a novel, implementable, and fast algorithm that can perform online optimal control tuning (OCTUNE) of a discrete linear time-invariant (LTI) controller in a classical feedback system, only using real-time system signals, i.e., no model required for the system under control. The OCTUNE algorithm uses the backpropagation optimization technique to optimize a performance function (squared error between the desired and actual signals) in real-time. Furthermore, convergence guarantees are derived using the Lyapunov stability theory to ensure stable tuning using online real-time data.

We demonstrate the effectiveness and practicality of the OCTUNE algorithm by applying it to the tuning of a discrete PID controller (a particular case of an LTI controller) that is used to stabilize the angular rates of a quadrotor unmanned aerial vehicle (UAV). The demonstration is performed in a realistic simulation environment using the Gazebo simulator and the robot operating system (ROS). The simulation results show how OCTUNE can be effectively used to automatically tune the UAV angular rate PID controllers using real-time signals in a fraction of a minute with a low number of online iterations. Finally, an open-source implementation of the OCTUNE algorithm is provided, which can be adapted for different applications.

The contributions of this work are summarized as follows.

- An online and model-free optimal auto-tuning algorithm for a generic LTI controller is developed, called OCTUNE, which is demonstrated using realistic simulations of a quadrotor system.
- Convergence proof of the OCTUNE algorithm is derived in order to guarantee safe control learning/tuning.
- We provide our implementation as an open-source software package of OCTUNE to facilitate the use and adaptation of the presented algorithm for different applications. The links to the open-source software is provided in the Supplementary Materials section.

The remainder of the paper is organized as follows. The problem statement is presented in Section 2. The optimal tuning algorithm is derived in Section 3, followed by convergence analysis in Section 4. Realistic simulation results of the OCTUNE algorithms for a quadrotor tuning application are discussed in Section 5. Finally, our conclusions and future work are discussed in Section 6.

## 2. Problem Statement

**Figure 1:**
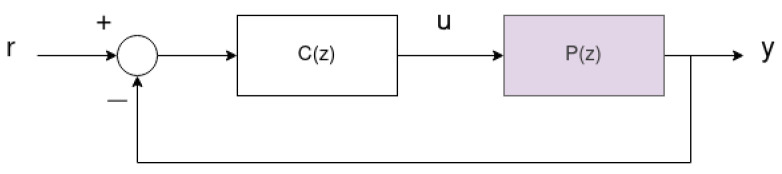
A feedback system with an unknown discrete-time plant, P(z), and a discrete-time LTI controller, C(z).

This section defines the controller architecture to be optimized using the OCTUNE algorithm described in Section 3 to improve the reference tracking in a classical feedback system. In this work, we assume a standard feedback system as shown in Figure 1, where the system is represented by an unknown discrete-time plant, P(z). The controller is assumed to be a discrete-time linear time-invariant (LTI) system of the following transfer function.

(1) $$C\left(z\right)=\frac{B(z)}{A(z)}=\frac{b_{0}+b_{1}z^{-1}+\cdots +b_{n_{b}}z^{-n_{b}}}{1+a_{1}z^{-1}+\cdots +a_{n_{a}}z^{-n_{a}}},$$

where *a*’s and *b*’s are the controller’s denominator and numerator coefficients, respectively. The system signals, reference r(k), controller output u(k), and output y(k) for time instances k=0,1,2,⋯ are all assumed to be measurable in real-time.

It is assumed that the controller in Equation (1) is initially stabilizing the feedback system in Figure 1. However, the performance defined later in Equation (2) may not be initially optimal. In other words, the system output y(k) is poorly tracking the reference r(k).

**Figure 2:**
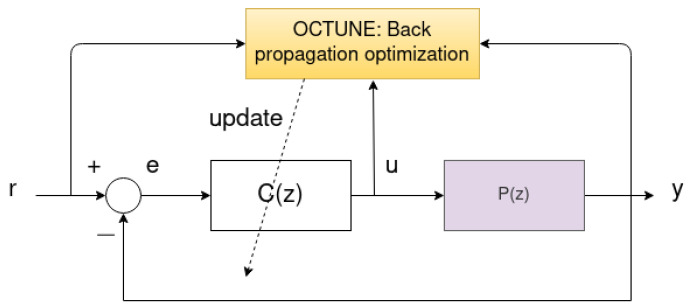
A feedback system with controller C(z) coefficients updated by the OCTUNE algorithm. The OCTUNE algorithm receives the reference, actual, and controller output signals and performs update operations to update the controller parameters.

The objective of this work is to find a controller structure of the form C(z), which optimizes the performance of the system response—defined later in Equation (2). To achieve this objective, we propose the OCTUNE algorithm, which updates the controller’s parameters *a* and *b* in real-time as shown in Figure 2. The OCTUNE block shown in Figure 2 receives r(k), y(k), and u(k) signals in real-time and computes the updated controller parameters in order to minimize the error between the reference signal r(k) and the actual output signal y(k). In addition, the OCTUNE algorithm updates the controller parameters while guaranteeing stable convergence to the minimum error using only real-time signals.

## 3. Control Tuning Algorithm

The objective of the OCTUNE is for the system output y(k) to track the desired reference signal r(k) as accurately as possible. Therefore, we define the objective function *L* that is to be optimized as follows.

(2) $$L\left(k\right)=\frac{1}{2}\sum_{i=k-N}^{k}e^{2}\left(i\right)$$

where *N* is the number of available data samples, and the error e(k) at time instant *k* is defined as the difference between the desired reference signal r(k) and the corresponding output signal y(k),

(3) e(k)=r(k)−y(k)

The objective function *L* can be written in a compact form as follows.

(4) $$L=\frac{1}{2}{\left||E|\right|}^{2}$$

where ||·|| is the Euclidean norm, and

(5) $$E={\left[e\left(k-N\right),e\left(k-N+1\right),\cdots ,e\left(k\right)\right]}^{T}$$

In the following section, an algorithm based on the backpropagation method is developed to compute the controller parameters in Equation (1) that minimize the objective function defined in Equation (4) given the system signals r(k),y(k), and u(k).

### Optimization Using Backpropagation

Backpropagation (BP) is a widely used algorithm in machine learning for efficiently training artificial neural networks (ANNs) [26]. BP computes the gradient (partial derivatives) of the loss function with respect to the weights of the neural network. The partial derivatives are then used to update the weight values. This process is repeated until convergence is achieved. The objective of this work is to compute the controller parameters (analogous to weights in ANNs) that minimize the loss function in Equation (2).

**Figure 3:**
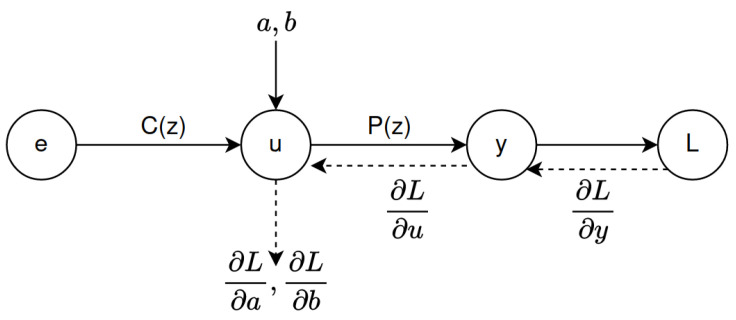
Forward and backward operations are used to compute the partial derivatives. Solid arrows represent forward propagation, and backpropagation is represented by dashed arrows.

As depicted in the computational graph in Figure 3, the backpropagation operations (represented using dashed lines) use the chain rule to compute the partial derivatives $\frac{\partial L}{\partial a},\frac{\partial L}{\partial b}$ through the intermediate partial derivatives $\frac{\partial L}{\partial y},\frac{\partial L}{\partial u}$. Then, the computed partial derivatives $\frac{\partial L}{\partial a},\frac{\partial L}{\partial b}$ are used to compute the new controller parameters a,b using the delta rule (gradient descent), Equation (16).

The optimization problem is defined as follows,

(6) $$\min_{a_{i},b_{i}}L=\frac{1}{2}\sum_{i=k-N}^{k}e^{2}\left(i\right)$$

In order to use the backpropagation algorithm [26] to solve (6), the partial derivatives of the objective function *L* with respect to the controller coefficients $a_{i},b_{i}$ need to be calculated, which is described as follows.

The objective *L* is directly a function of the error *e*; hence, the partial derivative of *L* with respect to the error $e_{k}$ at time *k*, and the error vector *E* at all *N* samples, are the first derivatives that need to be computed as follows.

(7) $$\begin{array}{cc} \frac{\partial L}{\partial e_{k}} & =e(k)\\ \frac{\partial L}{\partial E} & =E \end{array}$$

Next, going backward in the chain, the partial derivative of *L* with respect to the output $y_{k}$ at time *k* (and *y* for all *N* samples) is defined as follows,

(8) $$\begin{array}{cc} \frac{\partial L}{\partial y_{k}} & =\frac{\partial L}{\partial e_{k}}\frac{\partial e_{k}}{\partial y_{k}}=-e\left(k\right)\\ \frac{\partial L}{\partial y} & =-E \end{array}$$

Next, using the chain rule, the change of *L* with respect to the controller denominator coefficients $a_{i}$ is

(9) $$\begin{array}{cc} \frac{\partial L}{\partial a_{i}} & =\sum_{t=k-N}^{k}\frac{\partial L}{\partial e_{t}}\frac{\partial e_{t}}{\partial a_{i}}\\ \; & =\sum_{t=k-N}^{k}\frac{\partial L}{\partial e_{t}}\frac{\partial e_{t}}{\partial y_{t}}\frac{\partial y_{t}}{\partial u_{t}}\frac{\partial u_{t}}{\partial a_{i}}\\ \; & =\sum_{t=k-N}^{k}e\left(t\right)\left(-1\right)\frac{\partial y_{t}}{\partial u_{t}}\frac{\partial u_{t}}{\partial a_{i}}\\ i & =1,\cdots ,n_{a} \end{array}$$

Equation (9) can be written in a compact vector form as follows.

(10) $$\begin{array}{cc} \frac{\partial L}{\partial a} & =-J_{a}E\;\in R^{n_{a}}\\ J_{a} & =\left[\begin{array}{ccc} \frac{\partial y_{k-N}}{\partial u_{k-N}}\frac{\partial u_{k-N}}{\partial a_{1}} & \cdots & \frac{\partial y_{k}}{\partial u_{k}}\frac{\partial u_{k}}{\partial a_{1}}\\ \vdots & \vdots & \vdots \\ \frac{\partial y_{k-N}}{\partial u_{k-N}}\frac{\partial u_{k-N}}{\partial a_{n_{a}}} & \cdots & \frac{\partial y_{k}}{\partial u_{k}}\frac{\partial u_{k}}{\partial a_{n_{a}}} \end{array}\right]\in R^{n_{a}\times \left(N+1\right)} \end{array}$$

Similarly, the change of *L* with respect to the controller’s numerator coefficients $b_{j}$ can be calculated as follows.

(11) $$\begin{array}{cc} \frac{\partial L}{\partial b_{j}} & =\sum_{t=k-N}^{k}\frac{\partial L}{\partial e_{t}}\frac{\partial e_{t}}{\partial b_{j}}\\ \; & =\sum_{t=k-N}^{k}\frac{\partial L}{\partial e_{t}}\frac{\partial e_{t}}{\partial y_{t}}\frac{\partial y_{t}}{\partial u_{t}}\frac{\partial u_{t}}{\partial b_{j}}\\ \; & =\sum_{t=k-N}^{k}e\left(t\right)\left(-1\right)\frac{\partial y_{t}}{\partial u_{t}}\frac{\partial u_{t}}{\partial b_{j}}\\ j & =0,\cdots ,n_{b} \end{array}$$

Equation (11) can also be written in a compact vector form.

(12) $$\begin{array}{cc} \frac{\partial L}{\partial b} & =-J_{b}E\;\in R^{n_{b}}\\ J_{b} & =\left[\begin{array}{ccc} \frac{\partial y_{k-N}}{\partial u_{k-N}}\frac{\partial u_{k-N}}{\partial b_{0}} & \cdots & \frac{\partial y_{k}}{\partial u_{k}}\frac{\partial u_{k}}{\partial b_{0}}\\ \vdots & \vdots & \vdots \\ \frac{\partial y_{k-N}}{\partial u_{k-N}}\frac{\partial u_{k-N}}{\partial a_{n_{b}}} & \cdots & \frac{\partial y_{k}}{\partial u_{k}}\frac{\partial u_{k}}{\partial a_{n_{b}}} \end{array}\right]\in R^{n_{b}\times \left(N+1\right)} \end{array}$$

For compactness, let us define the following quantities, *W* is the controller parameter vector, *J* is the Jacobean matrix of all intermediate, data-driven, partial derivatives, and $\frac{\partial L}{\partial W}$ is the gradient vector of *L* with respect to the controller parameters *W*.

(13) $$W={\left[a_{1},\cdots ,a_{n_{a}},b_{0},\cdots ,b_{n_{b}}\right]}^{T}\in R^{n_{a}+n_{b}}$$

(14) $$J=\left[\begin{array}{c} J_{a}\\ J_{b} \end{array}\right]\in R^{\left(n_{a}+n_{b}\right)\times \left(N+1\right)}$$

(15) $$\frac{\partial L}{\partial W}=\left[\begin{array}{c} \frac{\partial L}{\partial a}\\ \;\\ \frac{\partial L}{\partial b} \end{array}\right]=\frac{\partial E}{\partial W}\frac{\partial L}{\partial E}=-J\cdot E\in R^{n_{a}+n_{b}}$$

Using Equation (15), the update rule of the controller coefficients $a_{i},b_{i}$ can be written as follows.

(16) $$\begin{array}{cc} W & W+\Delta W=W-\alpha \frac{\partial L}{\partial W}\\ \; & =W+\alpha J\cdot E \end{array}$$

The calculations of $\frac{\partial u}{\partial a_{i}}$ and $\frac{\partial u}{\partial b_{i}}$ are performed similar to the calculations of $\frac{\partial y}{\partial b_{j}}$ and $\frac{\partial y}{\partial a_{j}}$ in [20], and omitted here for brevity. In comparison to [20], in this work, we are identifying the coefficients of the controller’s transfer function instead of the plant’s.

In [20], the calculation of $\frac{\partial y}{\partial u}$, which is needed in Equations (9) and (11), was performed using the known linear structure of the plant P(z). However, in this work, this cannot be conducted in the same way as P(z) is assumed to be unknown. Instead, we assume that the system signals *y* and *u* are sampled fast enough, and the following first-order approximation is used.

(17) $$\frac{\partial y}{\partial u}\approx \frac{\Delta y}{\Delta u}=\frac{y(k)-y(k-1)}{u(k)-u(k-1)}$$

Algorithm 1 presents a pseudo code of the OCTUNE algorithm.

**Algorithm 1:**Pseudo code of the OCTUNE algorithm

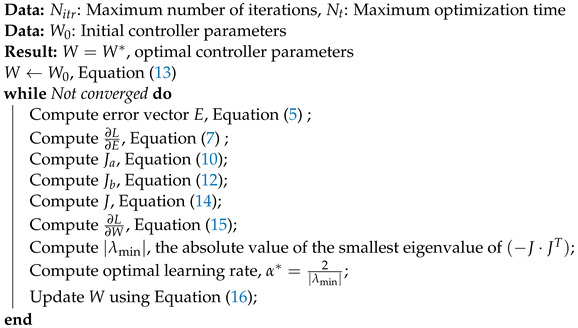



## 4. Convergence Analysis

The convergence of the controller coefficients $a_{i},b_{i}$ (or *W*) depends on the choice of the training rate α in Equation (16). High values of α can diverge the controller coefficients, while overly small values can guarantee convergence but with a slow training speed, which might not be practical for real-time applications. In this section, the procedure of selecting the proper values of α is developed.

Let V(k) be a discrete Lyapunov function [27] that is defined as follows.

(18) $$V\left(E\right)=\frac{1}{2}{\left||E|\right|}^{2}$$

where ||·|| is the 2-norm. The Lyapunov function V(E)=0 only when E=0. The change of *V*, ΔV is defined as follows.

(19) $$\begin{array}{cc} \Delta V & =V\left(E_{k+1}\right)-V\left(E_{k}\right)\\ \; & =\frac{1}{2}\left[\left|\right|E_{k+1}{\left|\right|}^{2}-\left|\right|E_{k}{\left|\right|}^{2}\right]\\ \; & =\Delta E^{T}\left[E_{k}+\frac{\Delta E}{2}\right] \end{array}$$

The error difference ΔE can be written as follows.

(20) $$E_{k+1}=E_{k}+\Delta E=E_{k}+{\left(\frac{\partial E_{k}}{\partial W_{k}}\right)}^{T}\cdot \Delta W_{k}$$

Using Equations (15) and (16),

(21) $$E_{k+1}=E_{k}-\alpha J^{T}JE_{k}$$

Therefore, ΔE can be defined as follows

(22) $$\Delta E=-\alpha J^{T}JE$$

**Theorem** **1.** *Let α be the learning rate used in the backpropagation algorithm in Equation (16) and $|\lambda_{\min}|$ be the absolute value of the smallest eigenvalue of $\left(-J\cdot J^{T}\right)$, where J is defined in Equation (14). Then, the convergence of the controller coefficients W is guaranteed if α is chosen such that it satisfies the following relationship.*

(23) $$0<\alpha <\frac{2}{|\lambda_{\min}|}$$

**Proof.**  Plugging Equation (22) into Equation (19) yields

(24) $$\begin{array}{cc} \Delta V & ={\left(-\alpha J^{T}JE\right)}^{T}\left[E+\frac{1}{2}\left(-\alpha J^{T}JE\right)\right]\\ \; & =\frac{-\alpha^{2}}{2}E^{T}J^{T}\left[\frac{2}{\alpha }I-JJ^{T}\right]JE \end{array}$$

From Equation (24), ΔV<0 if α>0 and $\frac{2}{\alpha }I-JJ^{T}$ is positive definite, which can be achieved by choosing α as in (23). With V(E)>0 for E≠0 and ΔV<0 satisfied by Equation (23), the convergence of *W* in Equation (16) is guaranteed. The optimal convergence can be achieved by $\alpha^{*}=\frac{2}{|\lambda_{\min}|}$. □

## 5. Validation: Quadrotor Tuning

This section presents realistic simulation results of the proposed OCTUNE algorithm applied to a practical use case of tuning a quadrotor’s PID angular rate control loops in real-time during flight. The angular rate stabilization is the innermost control loop and is the most critical one, which affects all the other higher control loops, such as the attitude, linear velocity, and position. For example, refer to the control architecture of the PX4 open-source autopilot PX4 control architecture [28].

Many UAVs use open-source autopilots, such as ArduPilot [4] and PX4 [3], in custom UAV research and development works. Usually, the custom-built UAVs that use off-the-shelf autopilots with open-source software, such as PX4 require iterative tuning of the PID control loops, which is generally performed manually before further development and flight testing. This manual process is essential to have a desirable flight performance. However, it can be cumbersome and time-consuming, as it requires manually performing flight tests, collecting data, manually analyzing them, and finally tuning the PID gains.

This manual tuning is conducted for each degree of freedom, i.e., three rotational (roll, pitch, and yaw) and three translational (x, y, and z) degrees, repeated many times until the desired control performance is achieved. In addition, a re-tuning process is needed if the UAV configuration is changed, for example, by adding or removing a payload. Moreover, the PID control loops might be tuned to work in specific environmental conditions, such as low wind speed. Therefore, it will need to be re-tuned to perform well against different disturbance sources and levels. An algorithm that can automatically and reliably tune controllers in such situations and in real-time is greatly beneficial as it saves time and optimizes performance.

The OCTUNE algorithm presented in this work effectively and practically addresses the above mentioned issues in real-time with no manual iterations or interventions. The OCTUNE algorithm is demonstrated with realistic quadrotor simulations in the following sections. A link to the video of the simulation experiments is provided in the Supplementary Materials section.

### 5.1. Simulation Setup

**Figure 4:**
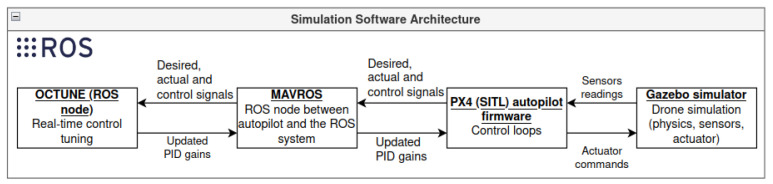
Abstract of the software architecture used in the simulations. Bold and underlined text represent software packages that are explained in Section 5.1.

The quadrotor simulation setup consists of the four main components depicted in Figure 4 and described as follows.

**Figure 5:**
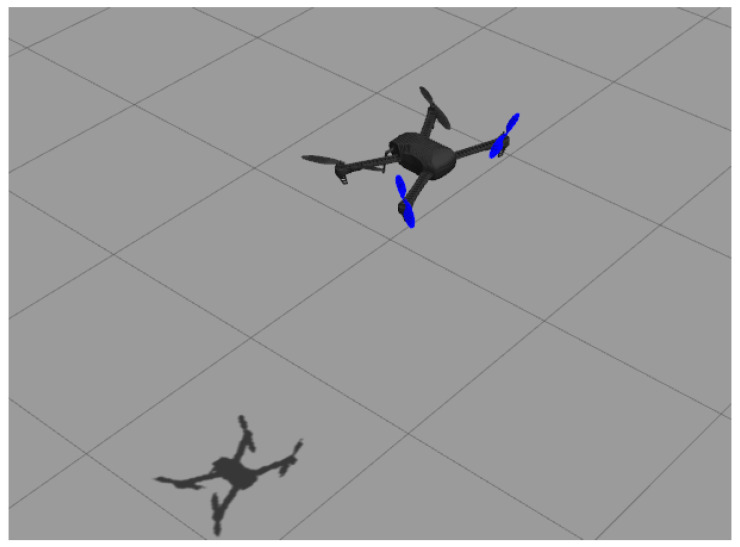
A snapshot of the Iris quadrotor model flying in the Gazebo simulator.

- Gazebo simulator: An open-source robot simulator [29] that accurately and efficiently simulates several types of robots in complex indoor and outdoor environments with multiple options of robust physics engines. It also has strong integration with the robot operating system (ROS) to facilitate software development and integration. The robot model simulated in this work is an actual quadrotor UAV called Iris; see Figure 5. The quadrotor model has several plugins to simulate the onboard sensors (Inertial measurement unit, GPS, and magnetometer) and the propulsion system. The model also models the mechanical structure of the drone with its mass and inertial characteristics.
- PX4 autopilot: This is the autopilot firmware that interfaces with Gazebo to receive the simulated sensors readings, to perform control and operations, and to send motor commands to the motor plugins of the Gazebo quadrotor model. The PX4 autopilot firmware implements the PID control loops tuned using the OCTUNE algorithm. The autopilot firmware in simulation (called software in the loop, SITL) is the same as the one on actual autopilot hardware, except that the actual sensors and motors are replaced with simulated ones.
- MAVROS: MAVROS is a software package that interfaces between the PX4 autopilot and the robot operating system (ROS) [30]. Interfacing PX4 with ROS makes the software development and integration extremely streamlined and can be easily deployed on actual hardware with almost no modifications to the software used in the simulation. The MAVROS communicates the required signals (target, controller output, and actual), and the PID controller gains between the OCTUNE application and the PX4 autopilot.
- OCTUNE: This is the implementation of the OCTUNE algorithm as a ROS software package (node in ROS terminology) for real-time tuning. The OCTUNE node receives the quadrotor signals (target, actual, and controller output), and the PID gains from the MAVROS node in real-time. After the signals and the current gains are used to compute the updated PID gains by the OCTUNE node, the new gains are sent to the PX4 autopilot via the MAVORS node.

### 5.2. Controller

The OCTUNE algorithm requires the definition of the controller’s transfer function as defined in Equation (1)—namely, the numerator coefficients $b_{i}$ and denominator coefficients $a_{i}$. An angular rate PID controller can be represented as a discrete-time transfer function [31] as follows.

(25) $$\begin{array}{cc} C\left(z\right)=\frac{U(z)}{E(z)} & =\frac{b_{0}+b_{1}z^{-1}+b_{2}z^{-2}}{1-z^{-1}}\\ b_{0} & =K_{p}+K_{d}/T+K_{i}T\\ b_{1} & =-2K_{d}/T-K_{p}\\ b_{2} & =K_{d}/T \end{array}$$

where $b_{0},b_{1}$, and $b_{2}$ are the controller’s transfer function numerator’s coefficients; $K_{p},K_{i}$, and $K_{d}$ are the proportional, integral, and differential PID gains; and *T* is the sampling time in seconds. With some algebraic manipulations, the PID gains can be calculated from the controller’s coefficients.

(26) $$\begin{array}{cc} K_{p} & =-2b_{2}-b_{1}\\ K_{i} & =\left(b_{0}+b_{1}+b_{2}\right)/\left(T\right)\\ K_{d} & =b_{2}/T \end{array}$$

### 5.3. Algorithm Implementation and State Machine

**Figure 6:**
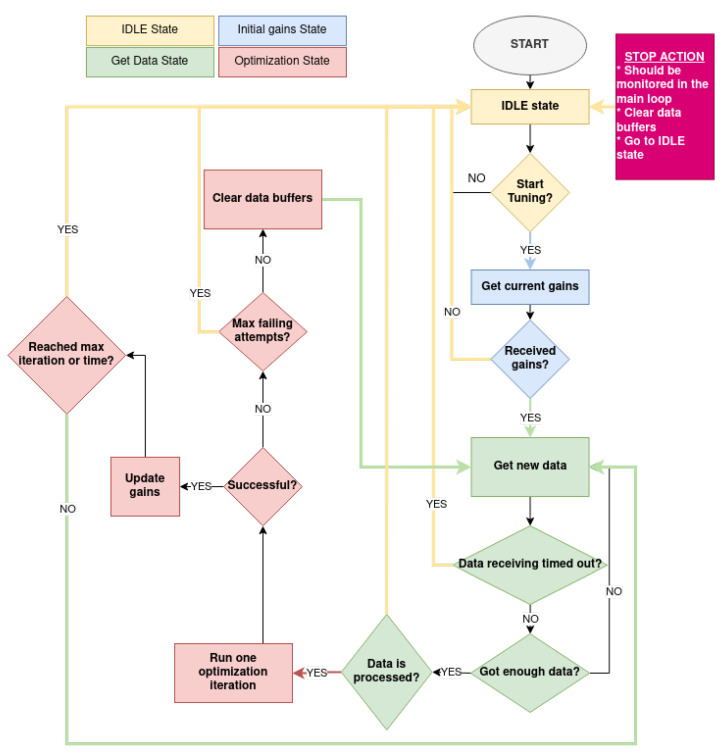
The auto-tuning state machine.

For the real-time safe implementation and execution of the OCTUNE algorithm, a state machine was designed to control the transitions between the different stages of the tuning process. The four primary states are depicted in detail in Figure 6 and described as follows.

- IDLE state: In the IDLE state, the tuning application waits for the operator to send a start signal. Upon receiving the start signal, the application transitions to the next state—the Get Data State.
- Initial Gain State: In this state, the initial (current) PID gains are requested from the autopilot. If there are no failures in receiving the initial gains, the application transitions to the next state, the Get Data Sate. Otherwise, it returns to the IDLE state.
- Get Data State: In this state, the required data for the tuning process, such as target, actual, and control output signals, are stored in buffers in real-time, over a predefined time period or number of samples. Once sufficient data samples are received, they are post-processed to align the data samples according to their time stamps and up-sampled to reduce the high-frequency noise in the acquired signals. If data post-processing is successfully performed, the application transitions to the next state—the Optimization state. Otherwise, the tuning process is stopped, and the application transitions to the IDLE state.
- Optimization state: In this state, an update step of the OCTUNE algorithm, Equation (16) is performed using the data collected and prepared in the Get Data State. The optimal learning rate $\alpha^{*}$ in Equation (23) is also computed in this state. If the update step is completed successfully, the application transitions back to the Get Data State to prepare a new set of signals for a new update iteration. If a termination condition is reached, such as the maximum optimization iteration or maximum optimization time, the state-machine is terminated, and the application transitions to the IDLE state to be ready for a new tuning cycle.

The aforementioned state-machine implementation is used to run multiple simulations of real-time tuning processes for the quadrotor system, which is discussed in the following sections.

### 5.4. Simulation Results with a Static Learning Rate, α

As mentioned, a primary contribution of this work is to guarantee stability during the tuning process in real-time, which is proved using the condition on the learning rate α, Equation (23). To demonstrate this, we compare the effect of executing the OCTUNE algorithm with a fixed learning rate α and with the optimal one in Equation (23) in simulations.

A simulation run was performed with a fixed learning rate α=0.001 for the angular rates of the roll and pitch PID control loops. In this simulation, the following steps were followed.

**Figure 7:**
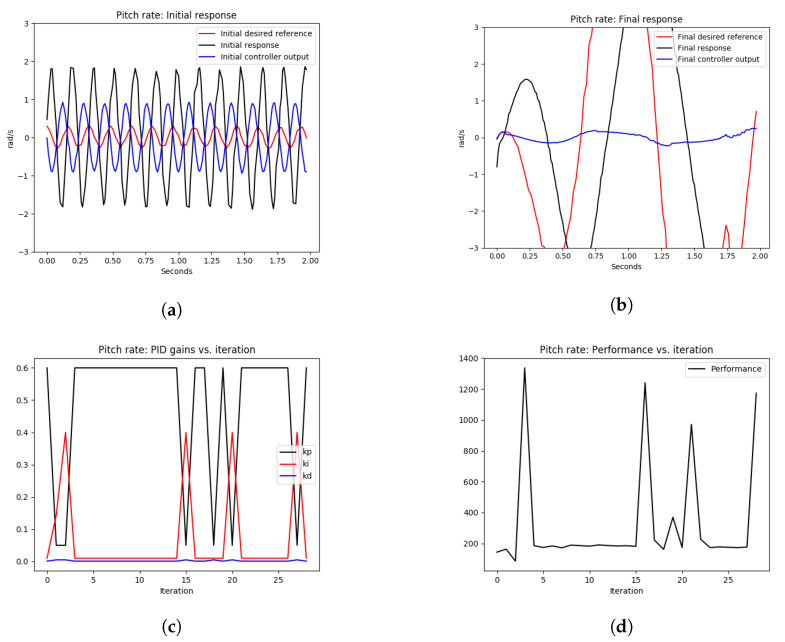
The pitch-rate tuning process during hovering. A fixed learning rate, α=0.001, was used. The quadrotor started with an oscillatory angular pitch response and ended with a worse response after tuning due to the use of a non-optimal fixed learning rate. (**a**) signals before tuning, (**b**) signals after tuning, (**c**) pitch rate PID gains, (**d**) performance error V(E) over iterations.

1 The quadrotor was commanded to take off in position stabilization mode and hover at 2 m above the ground. The quadrotor was initially stable.
2 The proportional gain of the pitch rate PID control loop was increased from 0.2 to 0.6 in order to introduce high-frequency oscillations.
3 The OCTUNE algorithm was started to tune the PID gains.
4 At the end of the tuning process, the tuning performance was shown using different plots as shown in Figure 7.

As shown in Figure 7a, the quadrotor initially had an oscillatory response in the angular rate control of the pitch axis due to high proportional gain; see the $K_{p}=0.6$ value at iteration 1 in Figure 7c. Since the learning rate α=0.001 was fixed over the entire optimization iterations, it resulted in the divergence of the system output in Figure 7b, the oscillatory behavior of the PID gains in Figure 7c, and non-diminishing performance error in Figure 7d. Therefore, using a fixed value of the learning rate α can be dangerous to the system tuning process as this cannot guarantee convergence.

In the next section, the simulations are performed with the optimal condition on the learning rate $\alpha^{*}$ to guarantee stability during the tuning process.

### 5.5. Simulation Results with an Optimal Learning Rate, $\alpha^{*}$

To guarantee the convergence of the performance metric *L* in Equation (4) of the angular rate control loops of the quadrotor system, the learning rate α was computed at each iteration, according to Equation (23), using the absolute value of the minimum eigenvalue of the Jacobean matrix *J* in Equation (14), which was constructed using the real-time signals, r(t), y(t), u(t).

Similar simulation steps were followed as in the static learning rate case, starting with the quadrotor in a hover state, increasing the proportional gain of both roll and pitch angular rate PID controllers to obtain high frequency oscillations, and finally starting the OCTUNE algorithm to tune the PID gains in using real-time simulated signals.

**Figure 8:**
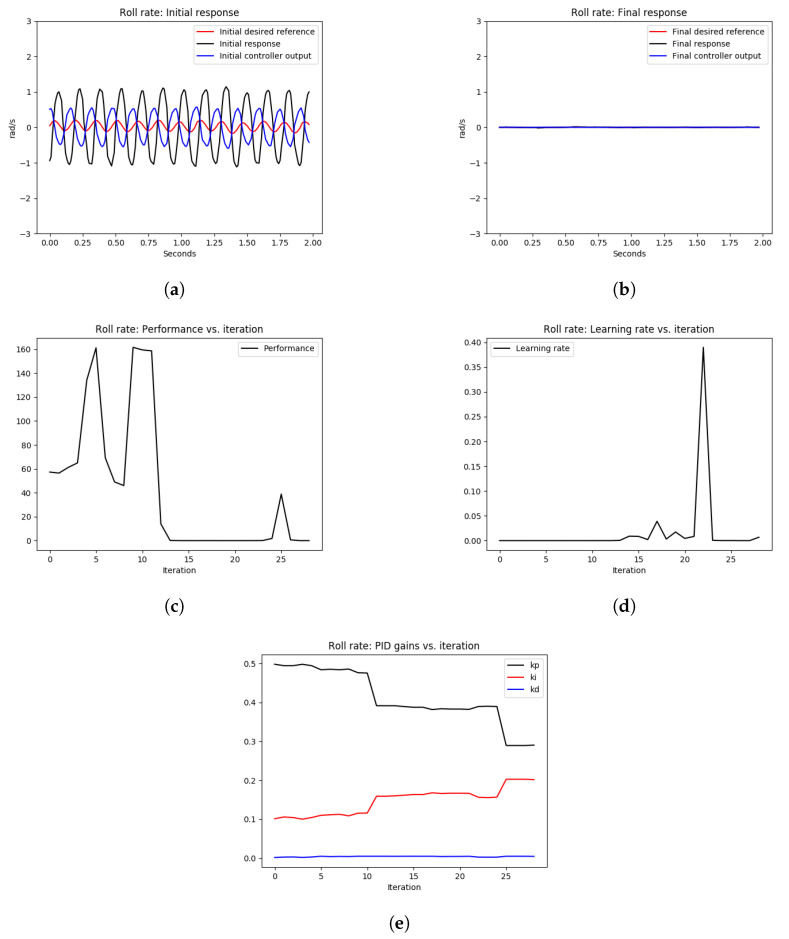
The tuning process of the roll-rate PID controller during hovering. The quadrotor starts with an oscillating behavior due to poorly tuned PID gains. Eventually, the angular rate loops are stabilized after the real-time tuning process. (**a**) signals before tuning, (**b**) signals after tuning, (**c**) performance error V(E) over iterations, (**d**) learning rate α over tuning iterations, (**e**) PID gains.

**Figure 9:**
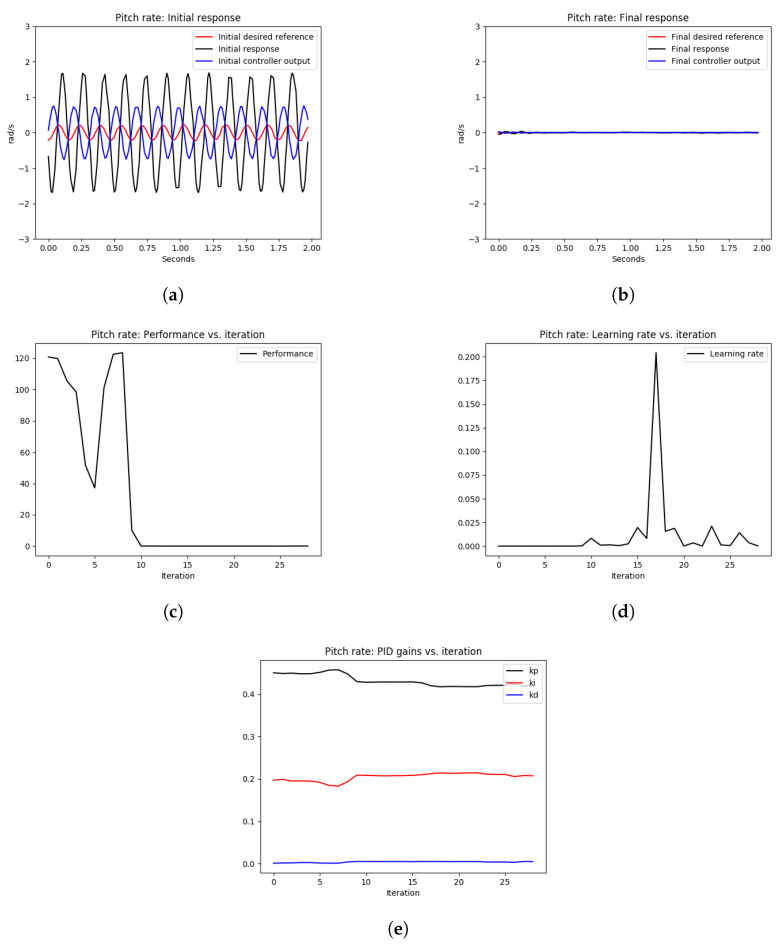
The tuning process of the pitch rate PID controller during hovering. The quadrotor starts with an oscillating behavior due to poorly tuned PID gains. Eventually, the angular rate loops are stabilized after the real-time tuning process. (**a**) signals before tuning, (**b**) signals after tuning, (**c**) performance error V(E) over iterations, (**d**) learning rate α over tuning iterations, (**e**) PID gains.

As shown in Figure 8 and Figure 9, the initial roll and pitch angular rate responses showed high-frequency oscillations due to the high proportional gains. After tuning the control loops using the OCTUNE algorithm over 28 iterations for 60 s, the control loops were stabilized as shown in Figure 8b and Figure 9b. In addition, the performance error L eventually diminished as shown in Figure 8c and Figure 9c.

In Figure 8d and Figure 9d, we can see that the learning rate α changes over iterations to guarantee that the performance error eventually converges. In comparison to the oscillating gains in Figure 7c, the gains in Figure 8e and Figure 9e are not oscillating and are tuned to reduce the performance error, which results in stable tracking of the angular rates as shown in Figure 8b and Figure 9b. The proportional gains are lowered to reduce the oscillations, and the integral gains are increased to reduce the steady-state error. The differential gains, however, have a minimal change, which is reasonable as high differential gains can cause system instability.

To provide numerical assessment of the tuning performance, we computed the mean squared error $\mathrm{MSE}=\frac{1}{n}\sum_{i=1}^{n}{\left(r\left(i\right)-y\left(i\right)\right)}^{2}$ before and after tuning. The number of data samples is constant in all experiments n=200, with time length T=2 seconds and the sampling rate dt=0.01.

Table 1 provides the mean squared error (MSE) of the simulation experiments of the pitch and roll-rate PID controllers, with an optimal learning rate $\alpha^{*}$ as depicted in Figure 8 and Figure 9. As shown in Table 1, the MSE for the roll rate after tuning is 5% of the MSE before tuning. Similarly, for the pitch rate control, the MSE after tuning is 0.82% of the MSE before tuning. This shows a significant improvement in the reference tracking of the rate PID control loops after the tuning process.

### 5.6. Hardware Experiments

**Figure 10:**
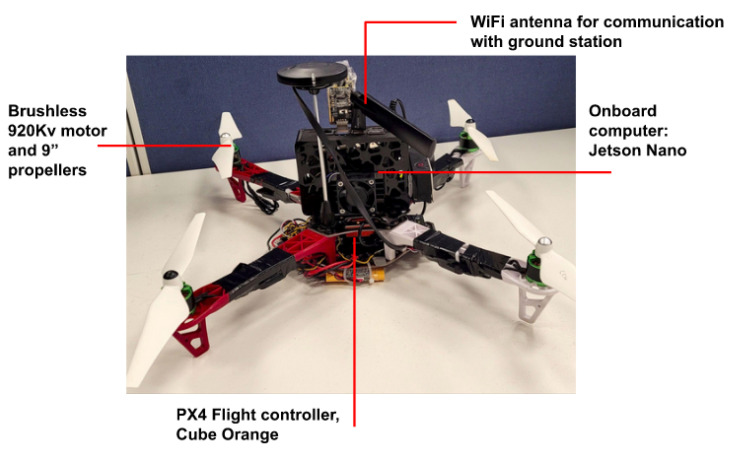
F450 quadcopter platform used in the OCTUNE hardware experiments.

This subsection provides validation results of the OCTUNE algorithm on a real quadcopter platform. The quadcopter used in the presented experiments is depicted in Figure 10. Three experiments were conducted in order to evaluate the OCTUNE performance under different initial PID gains. The PID controllers that were tuned in the hardware experiments were the same as the ones performed in simulation, which control the roll and pitch rates. The experiments were conducted in an indoor environment, and the quadcopter was controlled by a pilot. A link to the video of the hardware experiments are provided in the Supplementary Materials section. Each experiment’s design and results are presented as follows.

#### 5.6.1. Experiment 1

In this experiment, the PID gains of the roll and pitch rates were left at their default values, and the OCTUNE algorithm was executed during flight. The experiment steps are described as follows.

1 The drone is started on the ground with disarmed motors. The PID gains of the roll/pitch speed control loops are left at their default values (P=0.15,I=0.2, D=0.003).
2 The pilot flies the quadcopter to a hover position.
3 The OCTUNE process is started.
4 The pilot performs some maneuvers with the quadcopter in order to excite the system.
5 The OCTUNE process is stopped automatically after the indicated maximum optimization time, 120 s, is reached, and the logs and plots are saved.

**Figure 11:**
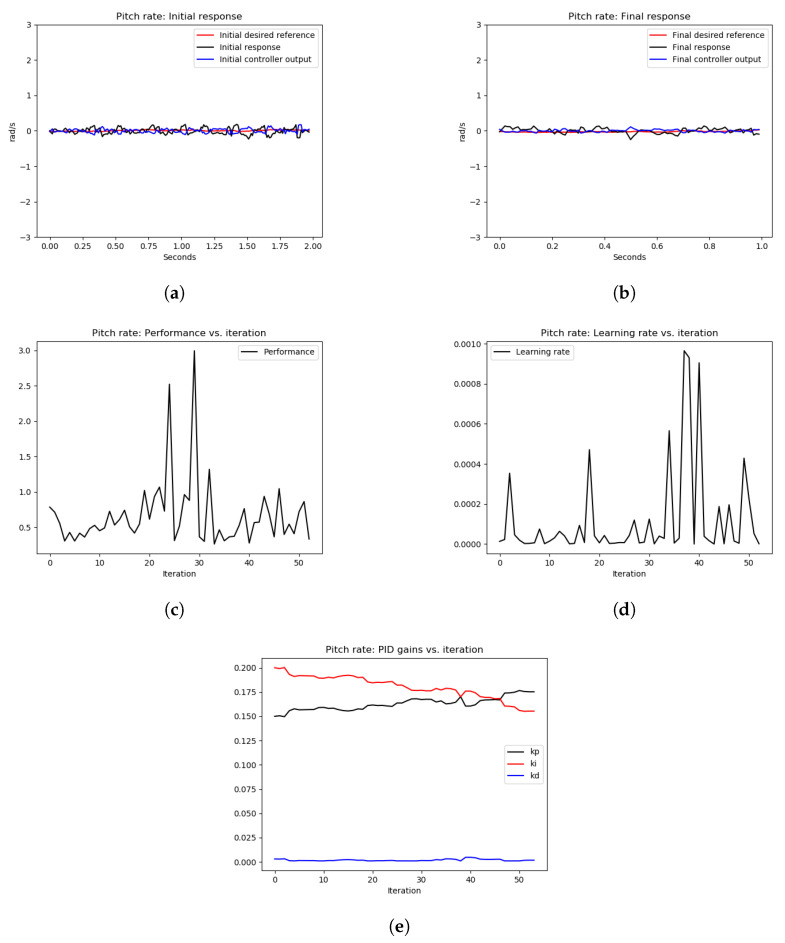
The results of the tuning process of the pitch rate PID controller in Experiment 1. (**a**) signals before tuning, (**b**) signals after tuning, (**c**) performance error V(E) over iterations, (**d**) learning rate α over tuning iterations, (**e**) PID gains.

**Figure 12:**
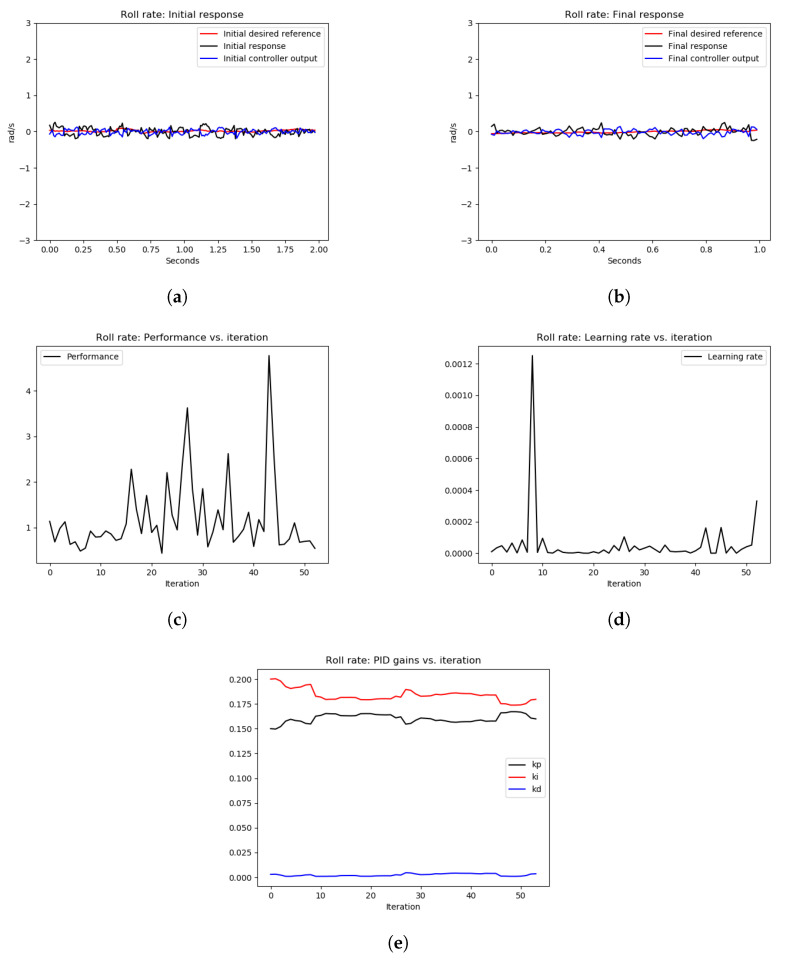
The results of the tuning process of the roll-rate PID controller in Experiment 1. (**a**) signals before tuning, (**b**) signals after tuning, (**c**) performance error V(E) over iterations, (**d**) learning rate α over tuning iterations, (**e**) PID gains.

As can be seen from Figure 11, the initial response as shown in Figure 11a and the final response as shown in Figure 11b show similar tracking performance. However, Figure 11e shows an increase in the P gain to have relatively faster tracking. Furthermore, the performance error in Figure 11c is small (≤3), which indicates acceptable tracking of the actual pitch rate signal to the desired one. This experiment demonstrates that starting for good PID gains that stabilize the system with good performance error, the OCTUNE algorithm does not drive the control system to an unstable state. It just improves its performance or at least maintains the current low-error performance. A similar observation of the roll axis can be seen in Figure 12.

#### 5.6.2. Experiment 2

In this experiment, the proportional gain (P) of the roll and pitch rates was increased dramatically, four times more than the default values (from 0.15 to 0.6, and, in order to introduce high-frequency oscillations, the OCTUNE algorithm was executed during flight, which should eventually tune the controllers to obtain rid of the oscillations. The experiment steps are described as follows.

1 Initially, the drone is on the ground, and the motors are disarmed. The PID gains of the roll/pitch speed control loops are left at their default values (*p* = 0.15, *I* = 0.2, *D* = 0.003).
2 The pilot flies the quadcopter to a hover position.
3 The P gains of the roll/pitch speed control loops are set to high values (from 0.15 to 0.6) to introduce high-frequency oscillations.
4 The OCTUNE process is started during the flight
5 The pilot tries to keep the quadcopter in hover position while tuning is running.
6 After the quadcopter stabilizes, the pilot performs some maneuvers with the quadcopter in order to excite the system and make sure the system is tuned well.
7 The OCTUNE process is stopped automatically after the indicated maximum optimization time, in the table below, is reached, and the logs and plots are saved.

**Figure 13:**
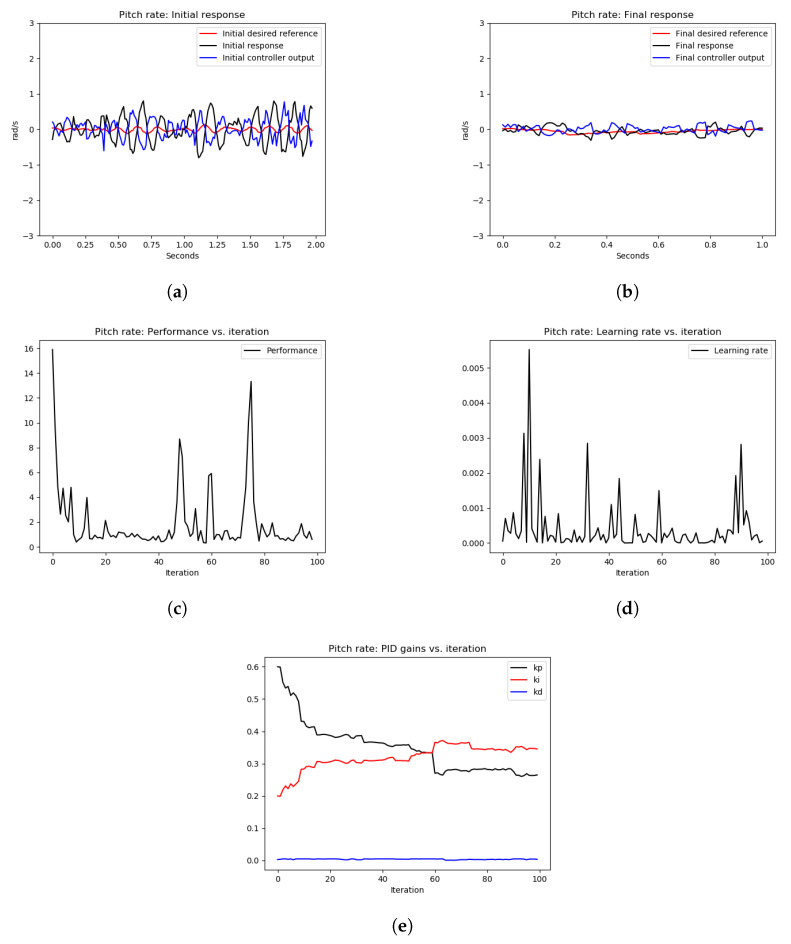
The results of the tuning process of the pitch rate PID controller in Experiment 2. (**a**) signals before tuning, (**b**) signals after tuning, (**c**) performance error V(E) over iterations, (**d**) learning rate α over tuning iterations, (**e**) PID gains.

**Figure 14:**
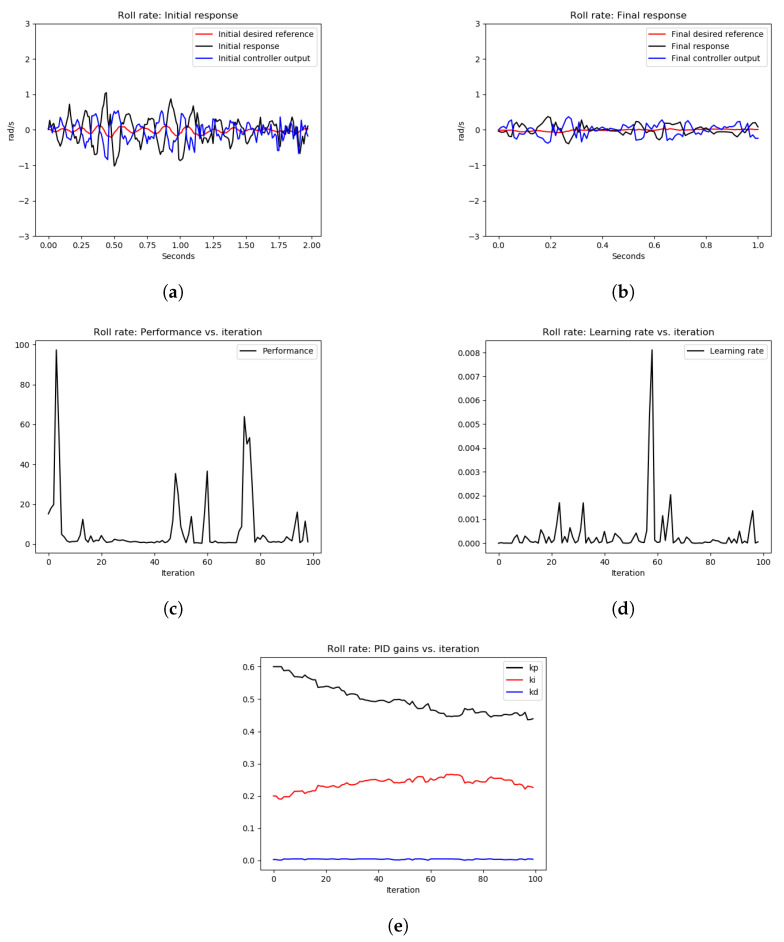
The results of the tuning process of the roll-rate PID controller in Experiment 2. (**a**) signals before tuning, (**b**) signals after tuning, (**c**) performance error V(E) over iterations, (**d**) learning rate α over tuning iterations, (**e**) PID gains.

The tuning results of Experiment 2 are depicted in Figure 13 and Figure 14, for pitch and roll axes, respectively. As can be seen from Figure 13a and Figure 14a, the initial response of the pitch and roll rates, respectively, show high-frequency oscillations as expected because the P gain of both controllers was increased dramatically. After executing the OCTUNE algorithm for 100 iterations (approximately 2 min), the performance error eventually decreased (see Figure 13c and Figure 14c) and the proportional gains were decreased as well (see Figure 13e and Figure 14e). As a result, the reference tracking is improved as shown in Figure 13b and Figure 14b.

Similar to the simulation experiments, we computed the MSE of the error signal in the hardware experiments before and after tuning. The MSE results are shown in Table 2. As shown in Table 2, the MSE of teh tracking error of the roll-rate PID controller after tuning is 5.9% of the MSE before tuning. Similarly, the MSE of tracking error of the pitch rate PID controller after tuning is 4.4% of the MSE before tuning. This shows significant improvement of the tracking performance after the tuning process in real-time.

## 6. Conclusions

In this paper, we presented the OCTUNE algorithm, which can be used for the optimal control tuning of an LTI controller (such as a PID) in a classical feedback system without the knowledge of the plant model and using only real-time signals.

The OCTUNE algorithm was validated in realistic simulations of a quadrotor UAV model and on a real quadrotor platform, in which the angular rates of PID controllers were stabilized in a fraction of a minute. The OCTUNE algorithm can run in real-time and continuously tune the controllers to account for any changes in the physical system (e.g., a change of payload) or environment (e.g., wind conditions), with proven convergence. In addition, an open-source implementation of the OCTUNE is available to facilitate the adaptation of the algorithm in different applications.

In future works, it would be interesting to generalize the OCTUNE algorithm to some nonlinear controllers with guaranteed convergence. Furthermore, the trade-off between robustness and optimality in real-time data-driven tuning is an exciting property to address.

## Figures and Tables

**Table 1:** The mean squared error (MSE) for the simulation results with the optimal learning rate, $\alpha^{*}$.

Experiment	MSE before Tuning	MSE after Tuning
Roll rate tuning	0.59	0.03
Pitch rate tuning	1.21	0.01

**Table 2:** The mean squared error (MSE) for hardware Experiment 2.

Experiment	MSE before Tuning	MSE after Tuning
Roll rate tuning	0.17	0.01
Pitch rate tuning	0.16	0.007

## Data Availability

Not applicable.

## Supplementary Materials

Supporting videos of simulations and hardware experiments, as well as the open-source codes, can be found in the following links: Video of the OCTUNE algorithm in quadcopter simulations: https://youtu.be/OY9XY9CdGhA; Video of the OCTUNE algorithm with actual quadcopter experiments: https://youtu.be/a3mrDvK2b-c; Open-source code of the OCTUNE algorithm: https://github.com/mzahana/octune; A ROS wrapper package to use OCTUNE with the PX4 flight controller: https://github.com/mzahana/px4_octune_ros. These links are accessed on 30 October 2022.

## Abbreviations

The following abbreviations are used in this manuscript:

OCTUNE	Optimal control tuning algorithm
PID	Proportional, integra, and derivative controller
UAV	Unmanned aerial vehicle
ROS	Robot operating system
